# Comprehensive genome analyses of *Sellimonas intestinalis*, a potential biomarker of homeostasis gut recovery

**DOI:** 10.1099/mgen.0.000476

**Published:** 2020-11-18

**Authors:** Marina Muñoz, Enzo Guerrero-Araya, Catalina Cortés-Tapia, Angela Plaza-Garrido, Trevor D. Lawley, Daniel Paredes-Sabja

**Affiliations:** ^1^​ Microbiota–Host Interactions and Clostridia Research Group, Departamento de Ciencias Biológicas, Facultad de Ciencias de la Vida, Universidad Andrés Bello, Santiago, Chile; ^2^​ ANID – Millennium Science Initiative Program – Millennium Nucleus in the Biology of the Intestinal Microbiota, Santiago, Chile; ^3^​ Host–Microbiota Interactions Laboratory, Wellcome Trust Sanger Institute, Wellcome Genome Campus, Hinxton, UK; ^4^​ Department of Biology, Texas A&M University, College Station, TX, 77843, USA

**Keywords:** *Sellimonas intestinalis*, phylogenomic, gut homeostasis, extremely oxygen-sensitive species

## Abstract

*
Sellimonas intestinalis* is a Gram-positive and anaerobic bacterial species previously considered as uncultivable. Although little is known about this *Lachnospiraceae* family member, its increased abundance has been reported in patients who have recovered from intestinal homeostasis after dysbiosis events. In this context, the aim of the present study was to take advantage of a massive *in vitro* culture protocol that allowed the recovery of extremely oxygen-sensitive species from faecal samples, which led to isolation of *S. intestinalis *. Whole genome analyses of 11 *S *. *intestinalis* genomes revealed that this species has a highly conserved genome with 99.7 % 16S rRNA gene sequence similarity, average nucleotide polymorphism results >95, and 50.1 % of its coding potential being part of the core genome. Despite this, the variable portion of its genome was informative enough to reveal the existence of three lineages (lineage-I including isolates from Chile and France, lineage-II from South Korea and Finland, and lineage-III from China and one isolate from the USA) and evidence of some recombination signals. The identification of a cluster of orthologous groups revealed a high number of genes involved in metabolism, including amino acid and carbohydrate transport as well as energy production and conversion, which matches with the metabolic profile previously reported for microbiota from healthy individuals. Additionally, virulence factors and antimicrobial resistance genes were found (mainly in lineage-III), which could favour their survival during antibiotic-induced dysbiosis. These findings provide the basis of knowledge about the potential of *S. intestinalis* as a bioindicator of intestinal homeostasis recovery and contribute to advancing the characterization of gut microbiota members with beneficial potential.

## Data Summary

The assembled genome obtained in this study was deposited at DDBJ/ENA/GenBank under accession number JACEEV000000000.

**Impact Statement**

The diversity and abundance of microbial species inhabiting the gut has been associated with health/disease events in multiple hosts. Although many of these species were considered uncultivable, this study makes use of an improved method of massive *in vitro* culture that allowed isolating and sequencing the genome of *Sellimonas intestinallis*, a species with potential as a marker of gut recovery homeostasis. Phylogenomic analysis performed for the first time for this species revealed at least three phylogroups that differentially carry antimicrobial resistance markers that could favour their survival under adverse conditions in the intestine.

## Introduction

The gut microbiota plays important roles in human and other mammalian species, including: (i) maintenance of the structural integrity of the intestinal epithelial barrier [1]; (ii) protection against the proliferation and colonization of enteropathogens [2]; (iii) metabolite production or conversion of substances for the host [3]; and (iv) stimulation of normal immune system functionality [4]. All these functions are determined by the diversity and abundance of microbial taxa that have been associated with host status (e.g. heath/disease, age, geographical origin among other comparison approaches) [5, 6]. Therefore, the scientific community has been focusing its efforts on deciphering the composition of the microbial communities that inhabit this ecosystem.

Classical techniques to detect and study microorganisms involve *in vitro* culture, but it is well known that most species inhabiting the human gut cannot be cultured under standard conditions [7]. To overcome this limitation, culture-independent DNA-based techniques, mainly based on next-generation sequencing (NGS), have been widely used to identify almost all species at the intestinal level. This is the case with targeted NGS which has become the most popular scheme to depict microbiota composition, thanks to the use of high-resolution markers to identify the taxonomic units (bacteria as well as eukaryotes and viruses), their variation among individuals or populations, and to infer phylogenetic relationships among the dominant taxa [8]. This approach has been complemented by shotgun metagenomics technology, which also allows the description of microbiota composition, and allows the assembly of whole genomes of the dominant taxa and the total content of nucleic acids present in the studied environment to be determined, which in the case of the gut, could provide informative markers of specific health/disease-promoting factors [9].

Studies based on culture-independent NGS have shown that *Ruminococcaceae* and *Lachnospiraceae* are the most abundant clostridial families in the gastrointestinal tract of humans and other mammals [10, 11]. Studies of species diversity and the role of these two families are ongoing, and changes in their relative abundance have been observed in dysbiosis, being positively associated with healthy groups [10]. In particular, the family *Lachnospiraceae* has gained interest in recent years due to the ecological adaptations exhibited by some of its species, associated with their ability to produce short-chain fatty acids (SCFAs) during glucose fermentation [12]. This capability attributed to commensal gut bacteria in healthy individuals has led to some *Lachnospiraceae* species as being proposed as potentially beneficial gut microbiota members [13]; however, few species of this family have been comprehensively studied.

One *Lachnospiraceae* species recently identified but poorly studied is *Sellimonas intestinalis *, a Gram-positive and obligately anaerobic bacterium [14], initially considered as part of the gut microbiota fraction that remains uncultivated owing to its extremely oxygen-sensitive (EOS) nature [15]. This limitation may explain the limited number of studies in which *S. intestinalis* has been detected, almost all of which aimed to decipher the microbiome composition from shotgun metagenomics approaches [15, 16]. In these studies, an increased relative abundance of *S. intestinalis* was detected in patients who recovered their intestinal homeostasis following dysbiosis caused by chemotherapy treatment for colorectal cancer [17] or therapeutic splenectomy of patients with liver cirrhosis [18]. These findings suggest the potential of *S. intestinalis* as a candidate biomarker of gut homeostasis recovery. Conversely, some transversal studies have detected an increased relative abundance of *S. intestinalis* in individuals with altered gut microbiota associated with chronic kidney disease [19] and systemic-onset juvenile idiopathic arthritis [20]. However, there have been no studies aimed at clarifying the role of *S. intestinalis* within the intestinal microbiome.

A pivotal step to clarify *S. intestinalis* in host gut homeostasis is to understand its genomic organization to identify the genetic basis of their ecological role. However, due to *in vitro* culture limitations, only eight draft genomes have been obtained as of November 2019 (https://www.ncbi.nlm.nih.gov/genome/genomes/41970), which were assembled from shotgun metagenomics data. These genomes have been reported mostly from eastern countries (China and South Korea), with a single genome reported from America (USA) [21].

For this reason, in this study we recover *S. intestinalis* using a massive *in vitro* culture approach directed to isolate oxygen-sensitive intestinal microbiota species. Subsequently, the isolate thus established was subject to whole genome sequencing and then included in a comprehensive whole genome analysis along with a set of 10 additional genomes publicly available for the species. The analysis scheme aimed to identify its genomic architecture, intra-taxon diversity, genetic population structure, potential metabolic profiles codifying for its genome and the presence of clinically important loci, as virulence factor markers (VFm) and antimicrobial resistance genes (AMRg), which could play a detrimental role in the colonization and relative abundance of this species in the complex intestinal environment. This approach represents an initial step to define the genomic bases that could support the role of this species in the intestinal microbiome and its potential as a biomarker of homeostasis gut recovery.

## Methods

### Sample collection

Stool samples were collected from adult Chilean individuals, within the framework of the project Millennium Nucleus in the Biology of Intestinal Microbiota. This project is aimed to detect and characterize the microorganisms that make up the intestinal microbiota of healthy individuals in Latin America. Each sample was collected in sterile containers with an airtight seal (to avoid direct exposure to oxygen) and without transport media [22].

### Bacterial isolate recovery

This study involved the optimization of a protocol for EOS intestinal bacteria isolation as follows: stool samples (collected in sterile containers without preservation media) were refrigerated (2–8 °C) and processed within the first 72 h after collection. Next, the samples were mechanically homogenized and divided into two fractions that were treated independently. The first (approximately 50 %) was washed with 100 % ethanol to reach 70 % (w/v) and incubated for 4 h under anaerobiosis. The biological material was then precipitated by centrifugation, to discard the ethanol, and then washed twice with sterile molecular-grade water. The second fraction of the sample was processed without washing. The two fractions were weighed, independently resuspended in sterile 1× PBS (1 ml per 100 mg of faeces) and then serially diluted (from 10^−1^ to 10^−5^ for the sample washed with ethanol and from 10^−1^ to 10^−8^ for the sample processed directly). Each dilution of the two treatments was seeded in duplicate on the complex and broad-range YCFA medium [23], in two formats: traditional or supplemented with taurocholate (Winckler) (0.1 %, v/v). Finally, they were incubated for 72–96 h at 37 °C under anaerobic conditions. The manipulation and incubation of samples were conducted in an anaerobic chamber (Bactron EZ2; ShellLab).

The colony-forming units (c.f.u.) obtained were streaked on YCFA plates, and after 24–48 h of incubation under the conditions described, their quality and morphology were evaluated by classical microbiological techniques (macroscopic and microscopic observation). The verified colonies were propagated in liquid YCFA medium to increase their biomass to establish the isolates, using the same incubation conditions. Although this approach led to the identification of a large number of colonies of different bacterial species, the isolate corresponding to *S. intestinallis* was obtained from a 24-year-old woman and was named 6K002.

### DNA extraction and whole genome sequencing (WGS)

The biomass recovered from isolate incubation in broth medium was subjected to DNA extraction using the commercial Wizard Genomic DNA Purification Kit (Promega), following the manufacturer’s recommendations. DNA sequencing was carried out by Wellcome Trust Sanger Institute on an Illumina HiSeq 2000 platform, with a read length of 100 bp, according to Dyke and Hubbard [24].

### Genome assembly and quality control verification

The reads obtained from WGS were *de novo* assembled using Unicycler v0.4.8, an assembly pipeline for bacterial genomes defined as a SPAdes-optimiser (Spades v3.13.1) which generates the best possible assembly [25], using default parameters. The quality of the genome assembly was evaluated using the GenomeQC_Filter_v1-5 script [26], which considers as parameters the maximum number of contigs per genome (fixed to 400) and a maximum size of each genome (considering 8 Mbp) and then extracts the small subunit 16S rRNA gene sequences (16S rRNA).

### Taxonomic placement and data retrieval

Initially, the 16S rRNA gene sequence previously extracted was used for sequence similarity searches against the data available in public datasets using the blastn algorithm [27], results that were subsequently verified by 16S rRNA gene sequence alignment using the silva Incremental Aligner (sina) service [28].

Next, a dataset with 2902 *Ruminococcaceae* and *Lachnospiraceae* genome assemblies, publicly available in the PATRIC [29, 30], ENA [31] and NCBI [32] databases and which passed the assembly quality test previously described, were analysed to identify the genomes most closely related to the analysed assembly. This dataset forms part of a parallel study by our research team directed to evaluate the phylogenetic relationships of the order *Clostridiales *. In parallel, a search of reads for the genus ‘*Sellimonas’* was conducted in the European Nucleotide Archive (https://www.ebi.ac.uk/ena/data/search?query=Sellimonas), with the aim of recovering the greatest number of genomes for analysis. The obtained reads were subject to the genome assembly and quality control verification methodology described in the previous section.

The complete genome dataset was used to select the node closely related to the analysed genomes, throughout phylogenetic reconstruction based on 16S rRNA gene sequences under the parameters described in the corresponding section. The set of assemblies selected was subjected to a step of delimiting species using average nucleotide identity (ANI) [33], using pyANI 0.2.10, a Python3 module and script that provides support for calculating ANI and related measures for whole genome comparisons, and rendering relevant graphical summary output (https://github.com/widdowquinn/pyani) [34]. pyANI analyses was developed using blast and other settings by default. Scores of ANI higher than 95.0% were used to verify that the genomes belong to the same species.

A graphical map of the genome assemblies identified as belonging to the same species as the studied genome was built in the CGview server [35], where a comparison was made in pairs to identify the differences between the genomes, using a tool based on the blast algorithm, included within the server.

### Annotation and pangenome analysis

An automated annotation pipeline was applied to the complete set of evaluated genomes. This pipeline is based on Prokka v1.13 [36], as follows: Infernal v1.1.2 [37] was run to predict RNA structures, followed by an analysis in Prodigal v2.6.3 [38] to predict proteins. Aragorn v1.2.38 [39] was used to predict tRNAs and tmRNAs, and Rnammer [27] was used to predict rRNAs. All predicted genes were then annotated throughout database searches in the following order: genus-specific databases were generated by retrieving the annotation from RefSeq [40]. The protein sequences were then merged using CD-hit version 4.8.1 [41] to produce a non-redundant blast protein database. Next, UniprotKB/SwissProt [42] was searched, considering kingdom-specific databases for bacteria. The complete set of genomes evaluated was subjected to the aforementioned annotation pipeline.

As a next step, the pangenome was determined using the Roary tool version 3.11.2 [43], taking as core genome definition a percentage identity of 95 % using Protein-Protein blast 2.9.0+ and presence in 99 % of the analysed genomes.

### Phylogeographical analyses

The phylogenetic relationships among *Ruminococcaceae* and *Lachnospiraceae* assemblies was evaluated to identify the data most closely related to the studied genome. For that, the 16S rRNA gene sequences extracted during the quality control verification step were aligned using MAFFT v7.407 [44] using default parameters and then an approximately maximum-likelihood phylogenetic tree was built in FastTree double precision version 2.1.10 [45] with default settings. The robustness of the nodes was evaluated using the bootstrap method (BT, with 1000 replicates).

After definition of the dataset to analyse, the phylogenetic relationships among isolates were evaluated using a Bayesian evolutionary approach based on Markov chain Monte Carlo (MCMC) implemented in Beast v1.10.4 [46] from the pangenome alignment (with a length of 22 453 nt) of the 11 sequence-selected assemblies. The GTR substitution model was chosen as the best model in jModelTest v0.1.1 [47], and an uncorrelated relaxed clock model and the skyline population model were considered as initial parameters. Twenty independent MCMC steps were carried out, each with a chain length of 100 000 000 states and resampling every 10 000 states. Log files were summarized with Tree Annotator v2.4.8 [44] using 10 % burn-in. The effective sample size (ESS) was >200 for all parameters; convergence and mixing were assessed using trace plot in Tracer v1.7.1 [48]. The tree files generated were summarized with Tree Annotator v2.4.8 [49] using 10 % burn-in, with maximum clade credibility and node heights at the heights of common ancestors. A node dating step was conducted using isolate metadata (date of isolate and geographical origin). A graphical visualization of all phylogenetic trees was obtained in the web tool Interactive Tree of Life v3 (http://itol.embl.de) [50]. Additionally, phylogenetic networks were conducted with the aim of detecting recombination signatures in the analysed population. These analyses were carried out in SplitsTree5 [51] using the neighbor-net method.

### Codifying potential of *S. intestinalis* genome

The annotation outputs were additionally used to identify Clusters of Orthologous Groups (COG) using eggNOG-mapper v2 under default settings, a tool for fast functional annotations of sequence collections [52]. The COG categories were subsequently were represented in a histogram.

VFm and AMRg were identified from whole genome assembles using Abricate 0.8.4 (https://github.com/tseemann/abricate), using blast searches against the sequences previously reported in the following databases: CARD (1749 sequences, last update: 8 July 2017) [53], Resfinder (1749 sequences, last update: 8 July 2017) [54], NCBI (1749 sequences, last update: 8 July 2017) [55], ARG-ANNOT (1749 sequences, last update: 8 July 2017) [56], VFDB (1749 sequences, last update: 8 July 2017) [57] and PlasmidFinder (1749 sequences, last update: 8 July 2017) [58]. A minimum DNA identity of 75 % was used as the detection threshold. As a confirmation step for VFm and AMR presence, Ariba (Antimicrobial Resistance Identification By Assembly) version 2.0 [59] was run from reads of the studied isolate.

## Results

### Biological source and isolation of *S. intestinalis* 6K002

A Gram-positive bacterial isolate with coccoid morphology (Fig. S1, available in the online version of this article) was established under the conditions to recover EOS microorganisms at the gastrointestinal level standardized by our research group. The biological source of this isolate was a stool sample from a 23-year-old woman who, despite being healthy at the time of sample collection, had a diagnosis of idiopathic rheumatoid arthritis. For this reason, she was under treatment with prednisone, a synthetic corticosteroid with glucocorticoid modulation, which provide its anti-inflammatory effect, and it has proven to be effective and safe for the treatment of patients with this pathology [60]. The individual was in addition taking *Chlorella* (microalgae containing omega-3 fatty acids and carotenoids with antioxidant effect that have been proposed as a potential source of renewable nutrition) [61], vitamin E with selenium and Korean ginseng. The individual was not on any antimicrobial treatment during the 6 months prior to sample collection.

### Assembly genome and taxonomic placement of *S. intestinalis* 6K002

**Fig. 1.**
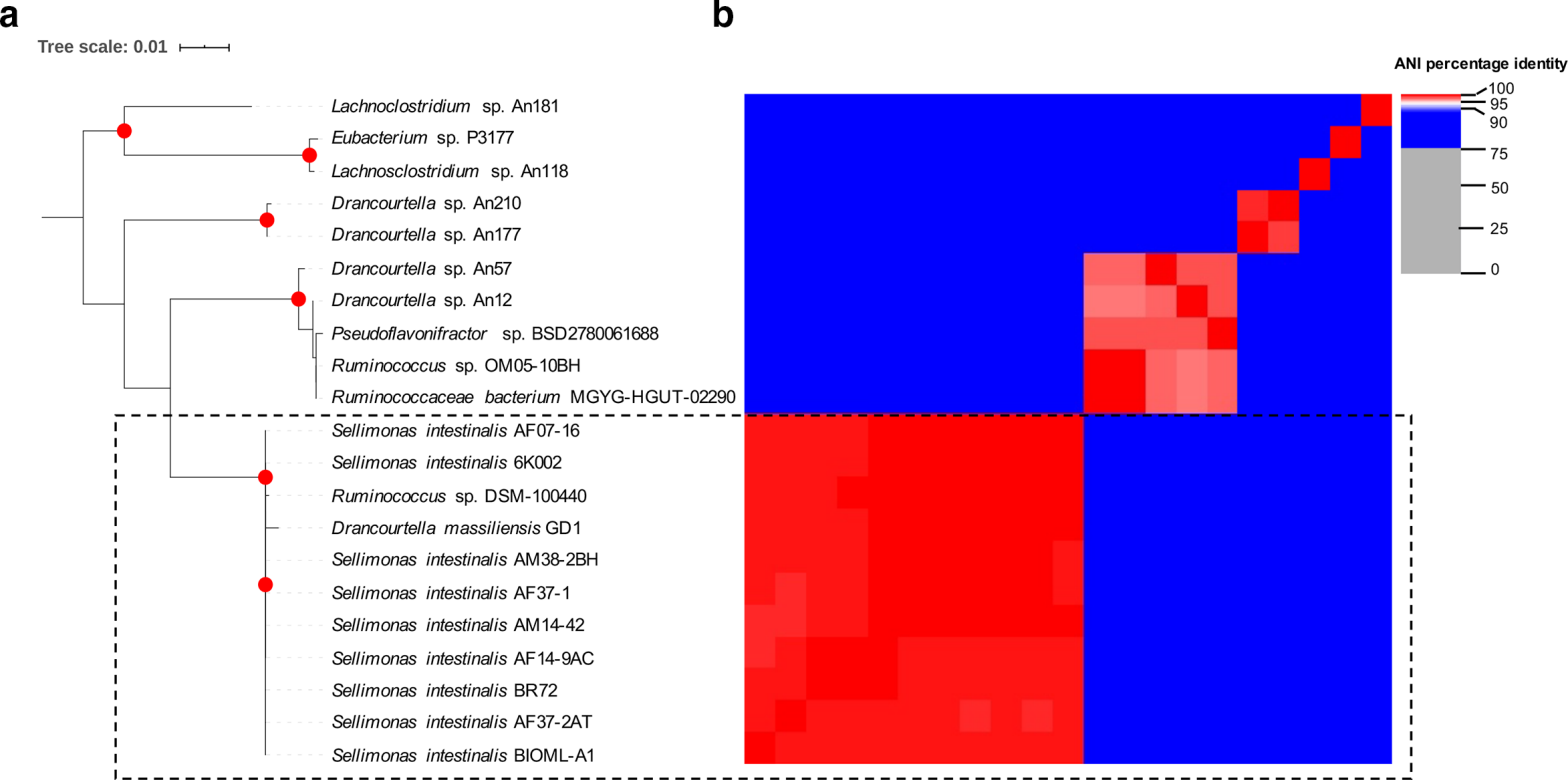
Taxonomic allocation analyses of the studied genome using a phylogenomic approach. (a) Phylogenetic reconstruction based on 16S rRNA gene alignment for the 21 selected genomes. Sequences were aligned using MAFFT [44] and then an approximately maximum-likelihood phylogenetic tree was built in FastTree double precision version 2.1.10 [45]. Interactive Tree of Life v3 (http://itol.embl.de) was used for graphical visualization [50]. Red dots represent bootstrap values ≥90.0. (b) ANI analysis for the selected dataset. Two genomes with ANI results >95 % are considered to belong to the same microbial species. The analysis was developed using pyANI (https://github.com/widdowquinn/pyani).

The assembled genome showed a length of 3 096 198 bp, constituted by 32 contigs with an N50 length of 439 526 bp, with 50 % of the sequence information in three large contigs. Extraction and subsequent comparison of the 16S rRNA gene sequence revealed that the analysed genome potentially belonged to one of the following genera: *Ruminococcus *, *Drancourtella* or *Sellimonas* (Table S1). The search of reads in the ENA database identified a report for one isolate that was assembled under the same conditions of the genome analysed in this study. The analysis of 2902 genomes reported as belonging to the families *Ruminococcaceae* and *Lachnospiraceae* (used during the preliminary analysis of data retrieval from the order *Clostridiales *) allowed us to identify that the analysed genome is part of a well-supported node that included 10 other genomes, most being reported as *Sellimonas intestinalis* (Fig. S2). These 11 genomes were then considered as the *S. intestinalis* node. Interestingly, two incongruencies in taxonomic allocation of publicly available genomes were detected, these being previously deposited as *Ruminococcus* sp. DSM-100440 and *Drancourtella massiliensis* GD1, and consistently clustered with the genome set under study [16S rRNA phylogenetic reconstruction (Fig. 1a) and ANI analysis (Fig. 1b)], that hereafter will be treated as part of the *S. intestinalis* node. This well-supported node was pruned to join the closest node (with seven genomes), that included mostly *Drancourtella* genomes, and was therefore identified as the *Drancourtella* node. Within this node were also found incongruences in taxonomic allocation, includingd two *Ruminococcus* and one *Pseudoflavonifractor* genomes (Fig. S2). Three additional representative genomes clustering in related nodes were included as outgroups (*Lachnosclostridium* sp. An181, *Eubacterium* sp. P3177 and *Lachnosclostridium* sp. An118). Under these parameters, a set of 21 assemblies were included in the data set for subsequent analysis.

The phylogenetic reconstruction based on 16S rRNA gene sequence alignment for the 21 selected genomes showed that the 11 genomes previously assigned to the *S. intestinalis* node remain clustered together (Fig. 1a) and had 99.7 % 16S rRNA gene sequence similarity. These findings were compared based on ANI, which was higher than 95 % for all these 11 *S *. *intestinalis* genomes (Fig. 1b), and verified that under the traditional phylogenetic criteria to identify microbial species (16S rRNA and ANI), all 11 assemblies correspond to *S. instestinalis* (Fig. 1a, b) . Information on the genomes included in *S. intestinalis* is given in Table S2.

### Identification of three potential main lineages of *S. intestinalis*

A preliminary blast comparison of 11 *S *. *intestinalis* selected assemblies revealed a high level of genome conservation; however, some genome regions were differentially present in groups of isolates. The map comparing the complete genomes delimited by these lineages is described in Fig. S3. As a next step, pangenome analysis of the *S. instestinalis* dataset showed a codifying potential of 4627 genes (Table S3), which are almost equally distributed between core genes (*n*=2318; 50.1 %) and accessory genes (*n*=2309; 49.9 %).

**Fig. 2.**
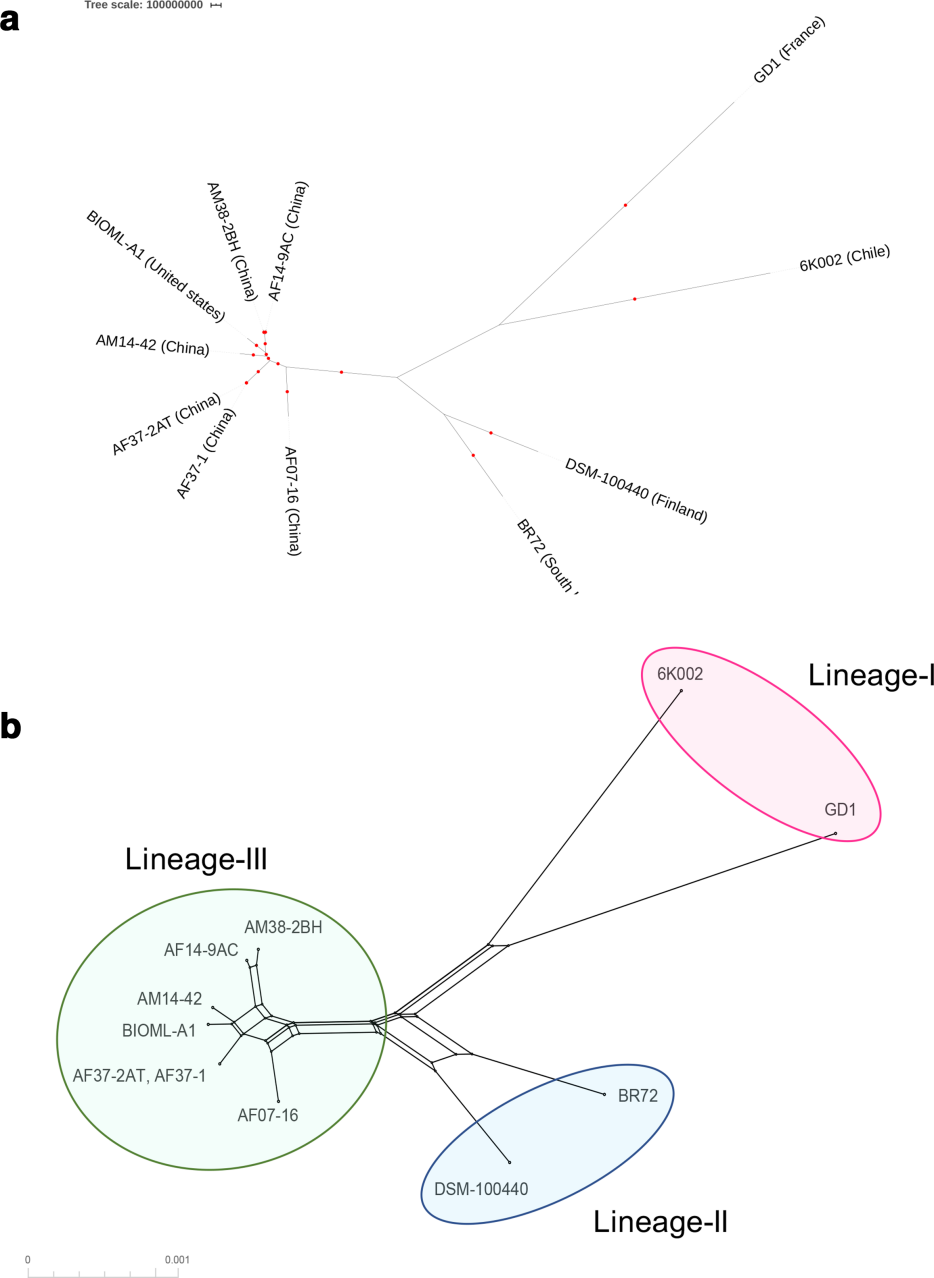
Phylogeographical analysis and phylogenetic networks used to predict the genetic population structure of *Sellimonas intestinalis *. (a) Bayesian evolutionary analysis based on MCMC implemented in beast-2 [49] carried out from the core genome alignment of the selected assemblies of the 11 sequences. The GTR substitution model was chosen as the best model in jModelTest v0.1.1 [47]. (b) Phylogenetic network using the neighbor-net method conducted in SplitsTree5 [51].

A phylogeographical analysis, based on a Bayesian evolutionary approach, was conducted from core genome alignment of the selected assemblies of the 11 sequences. Despite the limited number of genomes, the phylogenetic three topology revealed that *S. intestinalis* could have diversified into at least three major lineages with a possible relationship based on geographical origin (Fig. 2a). The first lineage (lineage-I) included isolates from Chile and France, while the second lineage (lineage-II) included isolates from South Korea and Finland, and the third lineage (lineage-III) included isolates mostly from China and only one from the USA. The phylogenetic network topology supported this population genetic structure, showing that although there are recombination signatures (indicated by observed reticulation events), the three lineages detected by phylogeographical analysis are divergent, supporting the hypothesis of the existence of three main populations within this species (Fig. 2b). Despite this clustering, a large distance was identified between the two strains belonging to lineage-I, even more than between lineage-II and III, which could suggest the existence of two sub-lineages within lineage-I.

### Metabolic profile of *S. intestinalis*

**Fig. 3.**
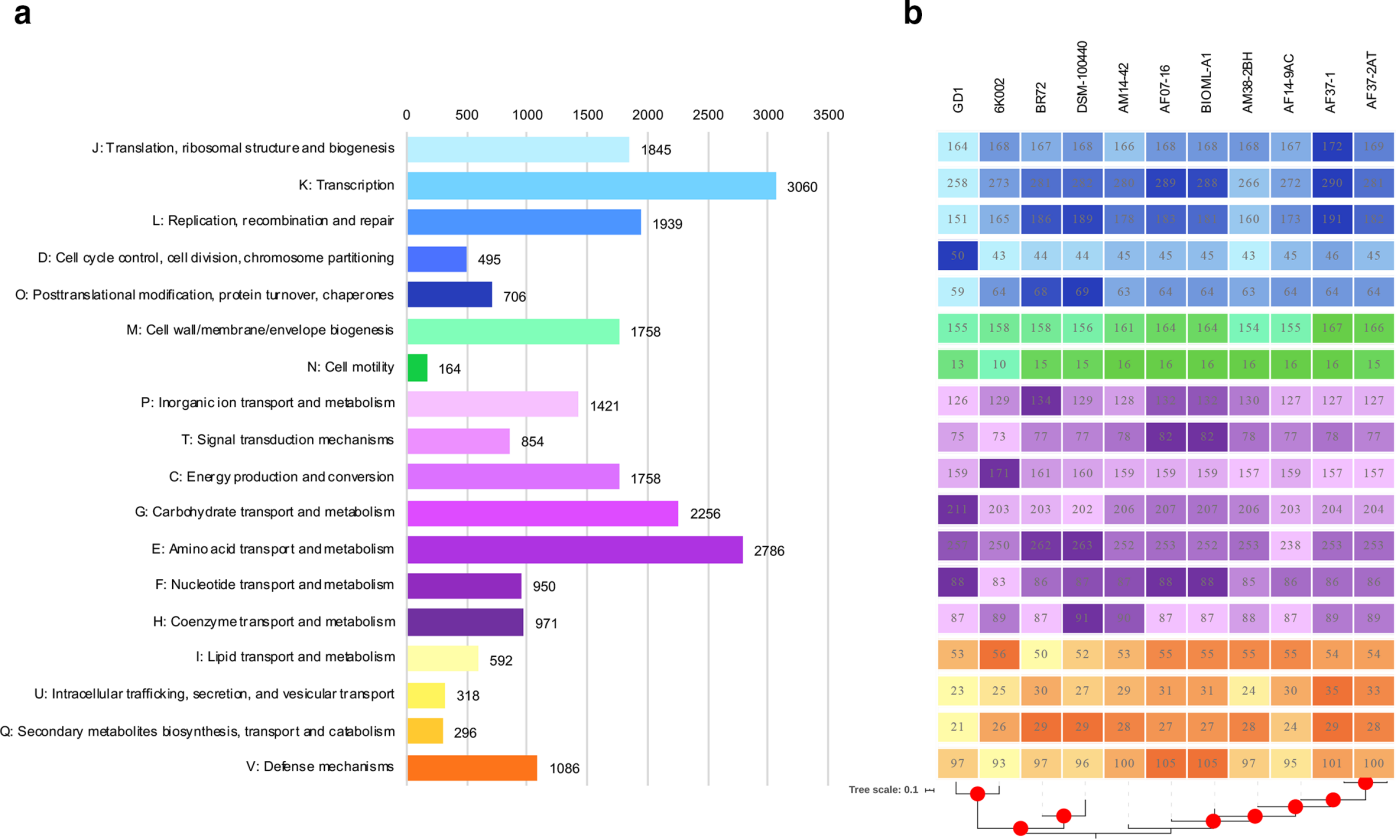
Clusters of Orthologous Groups (COGs) for (a) the global data set and (b) individual isolates. eggNOG-mapper v2 was used as a tool for fast functional annotations of sequence collections [52].

To explore the coding potential of the genome set under analysis, first COG set was developed for both the global data set (Fig. 3a) and individual isolates according to the lineages to which they belong (Fig. 3b). The results showed that this species directs much of the coding potential to essential biological processes such as transcription, translation and replication. However, it can be seen that an important part of their genes could be involved in metabolism, including amino acid and carbohydrate transport as well as energy production and conversion (Fig. 3a). There is no lineage-specific signal in the prevalence of genes attributed to different categories, although some differential profiles were detected in the identified populations, finding that the lineage-I and lineage-II clusters have more genes involved in metabolic processes, while lineage-III isolates had profiles with more genes involved in the cell cycle, intracellular trafficking, secretion and vesicular transport (Fig. 3b).

### *S. intestinalis* virulence factor and antimicrobial resistance encoding genes

**Fig. 4.**
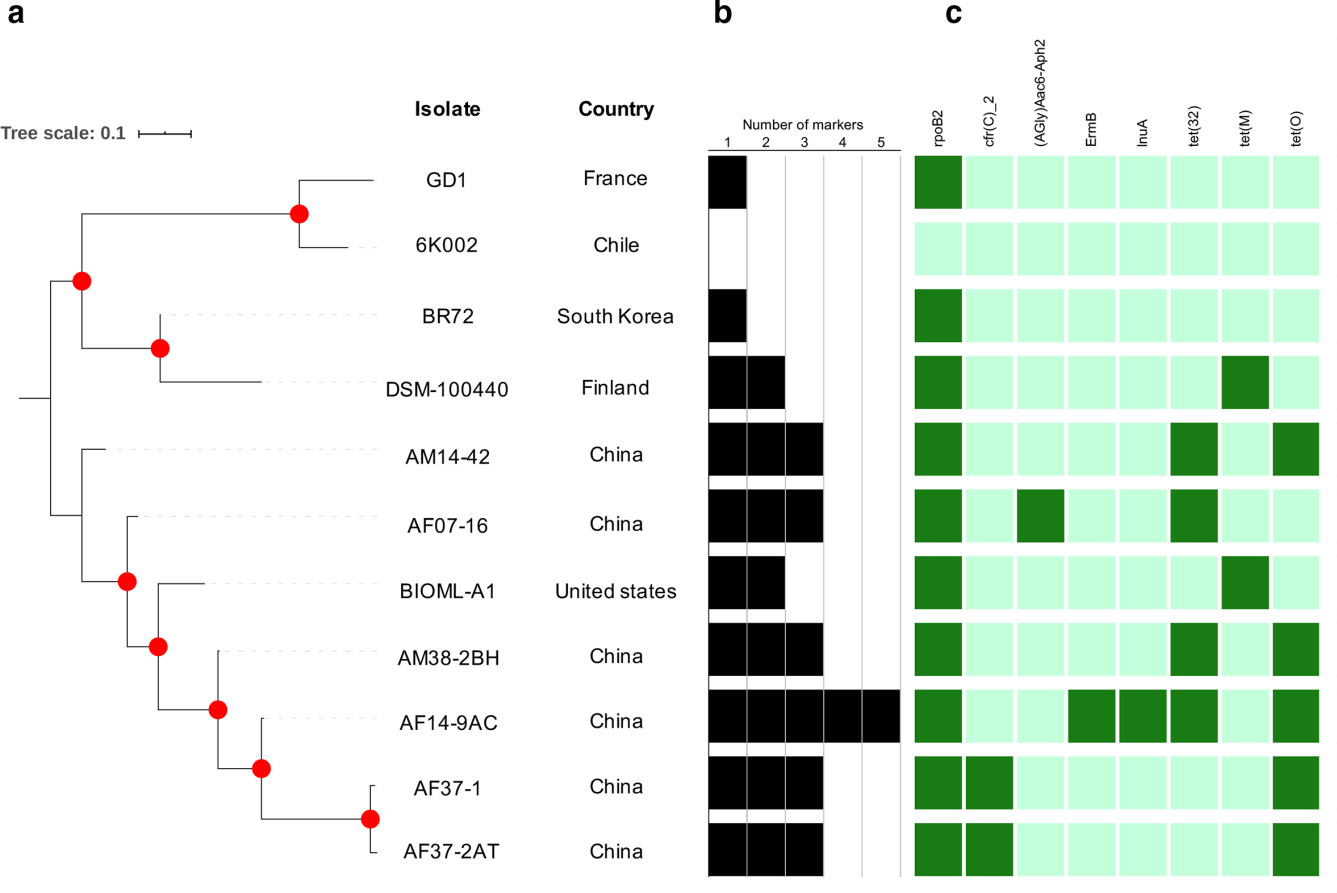
Virulence factors and antimicrobial resistance genes detected in *Sellimonas intestinalis* genomes. (a) Phylogenetic reconstruction from accessory genome alignment. (b) Frequency of markers found in each assembly. (c) Presence–absence matrix describing the markers detected in each genome. Abricate 0.8.4 (https://github.com/tseemann/abricate) was used to make blast searches against sequences previously reported in the following databases: CARD [53], Resfinder [54], NCBI [55], ARG-ANNOT [56], VFDB [57] and PlasmidFinder [58].

Given that about half of the genes coding for this species are part of the accessory genome, we inspected those genes differentially transported by the lineages detected (Fig. 4a). This analysis showed that the clustering in three populations is maintained in the phylogenetic reconstruction based on the accessory genome, as was found in the core genome phylogenetic analysis (Fig. 2a).

VFm and AMRg are important loci for survival of bacterial species because they can modulate changes in their abundance under different biological contexts, affecting the subsequent transmission dynamics between hosts (Fig. 4b). Although exhaustive search of both assemblies and reads revealed that the isolate from Chile (lineage-I) did not carry known VFm nor AMRg, an extended search of these loci from assemblies included in the comparative dataset revealed that the other genome clustered in the same lineage-I from France carrying the *rpoB2* marker, associated with resistance to rifampin resistance. *rpoB2* was found in all other nine evaluated genomes. The *tet*(M) marker (associated with tetracycline resistance) was the only additional marker found in lineage-II, being present in the isolate from Finland. Interestingly, lineage-III exhibited the greatest number of AMRg, with between two and five (in the case of AF14-9AC from China) genes per genome. Among the genes with higher frequency were: *tet* elements [*tet*32 and *tet*(O)], present in five and four genomes, respectively, and *cfr(C)_2* (conferring linezolid resistance) present in two genomes. In addition, (AGly)Aac6-Aph2, associated with aminoglycoside drug class resistance, *ermB* conferring macrolide–lincosamide–streptogramin antibiotic resistance, and *lnuA* associated with lincosamide resistance, all of these being present in a single genome each.

## Discussion

Recent studies using amplicon-based sequencing and shotgun metagenomics have contributed to the description of the diversity and abundance of gut microbial communities [62] and it has even been possible to propose associations with host states [63] and make inferences regarding the possible functions of specific members of this complex ecological network [64]. However, genomic characterization of gut microbiota members represents a challenge to deciphering the genetic bases supporting the biological function of microbial species inhabiting the gut, being an essential initial step in their recovery by *in vitro* culture, with an increased complexity for EOS species [65]. The present study describes the isolation and genomic features of *S. intestinalis *, a understudied *Lachnospiraceae* species recovered during a massive *in vitro* culture approach directed to recover EOS species within the microbiome environment.

During genomic characterization it is essential to have a precise taxonomic allocation of target genomes and those included in the comparative dataset to avoid mistakes with biological inference. In this study, inconsistencies in taxonomic classification were detected at different levels: (i) in the allocation of species to families with little phylogenetic relationship, as is the case of *Clostridium difficile* that had been included within the family *Clostridiaceae *, but after detailed analysis of the phylogenetic relationships was classified within the family *Peptostreptococcaceae* [66]; or (ii) in the taxonomic assignment of individuals, as revealed even before this work on *S. intestinalis *, which in other studies had previously been detected as *Ruminococcus* but with later sequencing of its complete genome was correctly assigned [15]. These types of findings reveal limitations in the traditional analysis schemes of complete genome data and underline the need for further studies to clarify the classification of under-studied anaerobic families.

The study of genetic population structure represents an important tool to determine the population sizes, dispersal potential and evolutionary rates over geographical scales during characterization of a microbial species [67]. For *S. intestinalis *, the low number of isolates analysed represents a limitation, although some remarkable profiles were identified, as is the case of the high degree of divergence between two members of lineage-I, which could suggest the existence of sub-lineages, and the slight degree of divergence between lineage-II and lineage-III showing a close relationship that could allow the exchange of genetic material, for example by recombination events (Fig. 2a, b). These findings were supported by pangenome results that revealed that despite the limited number of genes in the core genome (*n*=2318, 50.1 %), this could be a first indicator of the high intra-taxon diversity of this species. This type of finding has been detected in species such as *Pseudomonas aeruginosa* [68], a species of interest in health that exhibits a high frequency of gene loss and gain. The pangenome data also allowed the evaluation of phylogenetic relationships from core genome alignment (Fig. 2a), which led to the detection of three potential lineages. This clustering was subsequently confirmed through the construction of phylogenetic networks (Fig. 2b), which showed that despite the potential recombination events (supported by crosslinking in the networks), these three lineages could be highly divergent from each other. Interestingly, a possible common geographical origin was identified for lineage-III, whose members come mainly from China, with the exception of one isolate from the USA. The heterogeneous geographical origin of this last member of lineage-III and of the members of the other two lineages (I and II) could be attributed to human population migration, as has been identified for other pathogens [69]. However, the limited sample size is not enough to elucidate this hypothesis; further work with an increased number of individuals from different geographical origins will allow a more accurate picture of the population genetic structure of this species.

The effects of specific members of the gut microbiome have been attributed mainly to their metabolic profiling in which some sub-products can stimulate specific process in the complex gut environment [70]. The metabolic profiling of *S. intestinalis* from whole genome data using COG analysis was therefore able to decipher the genes required for the survival of the bacteria (Fig. 3a). At a general level, the genes associated with amino acid or carbohydrate transport as well as metabolism were highly frequent in this species. Analysis by isolate according to the lineage identified (Fig. 3b) showed that the isolates of oriental origin (lineage-III) codify a greater number of AMRg than the other geographical origins. These results are of importance, because these types of genes are determinants for metabolic processes that lead to the production of SCFAs during glucose fermentation [12], mainly butyric acid [71]. In the case of the patient studied in this work, the possible immunosuppression caused as a consequence of the anti-inflammatory effect of prednisone (corticosteroid used for the treatment of rheumatoid arthritis) [60] prescribed for idiopathic rheumatoid arthritis may have been balanced by: healthy lifestyle habits and/or the consumption of substances with potential restorative effects on the gut microbiota such as *Chlorella* (microalgae consumed by the patient because has potential as an antioxidant and in treatment of different health conditions) [61]. The presence of *S. intestinalis* could thus be in agreement with the hypothesis that it is a biomarker of the recovery of intestinal homeostasis.

The isolation and characterization of microbiota members contribute to deciphering the genomic bases of their effect in the gut microbial ecology [72], as well as to detect members that potentially play a role as reservoirs of antibiotic resistance [73]. In the particular case of *S. intestinalis *, several genes associated with antibiotic resistance were found, the most frequent being *rpoB2* conferring resistance to rifampin [74], which was present in ten of the 11 analysed genomes. Other highly frequent markers were the mobile genetic elements *tet* (O), (32) and (M), present in five, three and two genomes respectively (Fig. 4). Recently it has been proposed that antimicrobial activity has improved the quality of life and increased the life expectancy of microorganisms inhabiting the human gut [75], so the ability of *S. instestinalis* to carry AMRg could represent the basis for the survival of this species at the intestinal level, despite the adverse conditions that this niche naturally represents or under disruption events. It could explain the role of this species as a biomarker of homeostasis gut recovery, after presentation and restoration of homeostasis after dysbiosis generated by different causes. However, a limitation of our current work is that we did not conduct an *in vitro* test to identify the minimum inhibitory concentrations at which the proliferation of this species is inhibited. Further experiments will consider the antibiotic resistance profile of a group o*f S. intestinalis* strains to further explore this important trait.

Given the differential presence of genes that are biologically and clinically relevant among the three *S. intestinalis* lineages found in this work, future studies are needed to develop a molecular typing method to quickly identify isolates and which contributes to clarifying the phylogenetic relationships and evolutionary history of this species. Additionally, taking into account that this study aimed to analyse whole genome data of *S. intestinalis* and no phenotypic tests were performed, it is necessary to carry out further studies, including the determination of minimal inhibitory concentration, that can identify the potential role of VFm and AMRg and their modulation of the relative abundance of this species under different biotic contexts. Despite this limitation, the identification of these markers could support the hypothesis that some members of the microbiota could fulfil a resistance reservoir function, from which bacterial pathogens can acquire resistance in the human gut microbiota [15], and of interest at the health level.

This study represents the first step to decipher the genetic bases of the potential beneficial effect of *S. instestinalis* to re-establishment of gut homeostasis. Furthermore, the identification of AMRg suggests a mechanism possibly involved in the survival of this microorganism under antibiotic-induced dysbiosis. Despite the contribution of this work to advance our knowledge of this under-studied species, we additionally needto: (i) evaluate its relative abundance through microbial ecology studies in populations from different geographical origins and with heterogeneous health states; (ii) evaluate survival and effect on intestinal homeostasis through longitudinal studies, initially in animal models and then in human populations; and, most importantly, (iii) carry out *in vitro* antimicrobial resistance tests that lead to clarifying the phenotypic effect of genes encoding the genome of this species, particularly AMRg. Together, such studies would contribute to the identification of a new generation of probiotics with potential use in the recovery of intestinal homeostasis.

## Supplementary Data

Supplementary material 1

## References

[1] Barko PC, McMichael MA, Swanson KS, Williams DA (2018). The gastrointestinal microbiome: a review. J Vet Intern Med.

[2] Rinninella E, Raoul P, Cintoni M, Franceschi F, Miggiano GAD (2019). What is the healthy gut microbiota composition? a changing ecosystem across age, environment, diet, and diseases. Microorganisms.

[3] Blaut M (2013). Ecology and physiology of the intestinal tract. Curr Top Microbiol Immunol.

[4] Eberl G (2018). The microbiota, a necessary element of immunity. C R Biol.

[5] Cong J, Zhang X (2018). How human microbiome talks to health and disease. Eur J Clin Microbiol Infect Dis.

[6] Gupta VK, Paul S, Dutta C (2017). Geography, ethnicity or Subsistence-Specific variations in human microbiome composition and diversity. Front Microbiol.

[7] Carabeo-Pérez A, Guerra-Rivera G, Ramos-Leal M, Jiménez-Hernández J (2019). Metagenomic approaches: effective tools for monitoring the structure and functionality of microbiomes in anaerobic digestion systems. Appl Microbiol Biotechnol.

[8] Fraher MH, O'Toole PW, Quigley EMM (2012). Techniques used to characterize the gut microbiota: a guide for the clinician. Nat Rev Gastroenterol Hepatol.

[9] Mitchell SL, Simner PJ (2019). Next-Generation sequencing in clinical microbiology: are we there yet?. Clin Lab Med.

[10] Lozupone CA, Stombaugh J, Gonzalez A, Ackermann G, Wendel D (2013). Meta-Analyses of studies of the human microbiota. Genome Res.

[11] Suchodolski JS (2011). Intestinal microbiota of dogs and cats: a bigger world than we thought. Vet Clin North Am Small Anim Pract.

[12] Meehan CJ, Beiko RG (2014). A phylogenomic view of ecological specialization in the Lachnospiraceae, a family of digestive tract-associated bacteria. Genome Biol Evol.

[13] Brestoff JR, Artis D (2013). Commensal bacteria at the interface of host metabolism and the immune system. Nat Immunol.

[14] Seo B, Yoo JE, Lee YM, Ko G (2016). *Sellimonas intestinalis* gen. nov., sp. nov., isolated from human faeces. Int J Syst Evol Microbiol.

[15] Versluis D, de J Bello González T, Zoetendal EG, Passel MWJvan, Smidt H (2019). High throughput cultivation-based screening on porous aluminum oxide chips allows targeted isolation of antibiotic resistant human gut bacteria. PLoS One.

[16] Sun Y, Chen Q, Lin P, Xu R, He D (2019). Characteristics of gut microbiota in patients with rheumatoid arthritis in Shanghai, China. Front Cell Infect Microbiol.

[17] Kong C, Gao R, Yan X, Huang L, He J (2019). Alterations in intestinal microbiota of colorectal cancer patients receiving radical surgery combined with adjuvant CapeOx therapy. Sci China Life Sci.

[18] Liu Y, Li J, Jin Y, Zhao L, Zhao F (2018). Splenectomy leads to amelioration of altered gut microbiota and metabolome in liver cirrhosis patients. Front Microbiol.

[19] Lun H, Yang W, Zhao S, Jiang M, Xu M (2019). Altered gut microbiota and microbial biomarkers associated with chronic kidney disease. Microbiologyopen.

[20] Dong Y-Q, Wang W, Li J, Ma M-S, Zhong L-Q (2019). Characterization of microbiota in systemic-onset juvenile idiopathic arthritis with different disease severities. World J Clin Cases.

[21] Poyet M, Groussin M, Gibbons SM, Avila-Pacheco J, Jiang X (2019). A library of human gut bacterial isolates paired with longitudinal multiomics data enables mechanistic microbiome research. Nat Med.

[22] Siah SP, Merif J, Kaur K, Nair J, Huntington PG (2014). Improved detection of gastrointestinal pathogens using generalised sample processing and amplification panels. Pathology.

[23] Browne HP, Forster SC, Anonye BO, Kumar N, Neville BA (2016). Culturing of 'unculturable' human microbiota reveals novel taxa and extensive sporulation. Nature.

[24] Dyke SO, Hubbard TJ (2011). Developing and implementing an institute-wide data sharing policy. Genome Med.

[25] Wick RR, Judd LM, Gorrie CL, Holt KE (2017). Unicycler: resolving bacterial genome assemblies from short and long sequencing reads. PLoS Comput Biol.

[26] Gualtero SM, Abril LA, Camelo N, Sanchez SD, Davila FA (2017). [Characteristics of Clostridium difficile infection in a high complexity hospital and report of the circulation of the NAP1/027 hypervirulent strain in Colombia]. Biomedica.

[27] Lagesen K, Hallin P, Rødland EA, Staerfeldt H-H, Rognes T (2007). RNAmmer: consistent and rapid annotation of ribosomal RNA genes. Nucleic Acids Res.

[28] Quast C, Pruesse E, Yilmaz P, Gerken J, Schweer T (2013). The Silva ribosomal RNA gene database project: improved data processing and web-based tools. Nucleic Acids Res.

[29] Wattam AR, Abraham D, Dalay O, Disz TL, Driscoll T (2014). PATRIC, the bacterial bioinformatics database and analysis resource. *Nucleic Acids* Res.

[30] Wattam AR, Abraham D, Dalay O, Disz TL, Driscoll T (2017). Improvements to PATRIC, the all-bacterial Bioinformatics Database and Analysis Resource Center. PATRIC 3.5.4. Search criteria: Genomes/Clostridium. https://www.patricbrc.org/view/GenomeList/?keyword(clostridium)#view_tab=genomes&filter=eq(genome_status,%22Complete%22. https://www.patricbrc.org/view/GenomeList/?keyword(clostridium)#view_tab=genomes&filter=eq(genome_status,%22Complete%22.

[31] Silvester N, Alako B, Amid C, Cerdeño-Tarrága A, Clarke L (2018). The European nucleotide Archive in 2017. Nucleic Acids Res.

[32] Wheeler DL, Chappey C, Lash AE, Leipe DD, Madden TL (2000). Database resources of the National center for biotechnology information. Nucleic Acids Res.

[33] Figueras MJ, Beaz-Hidalgo R, Hossain MJ, Liles MR (2014). Taxonomic affiliation of new genomes should be verified using average nucleotide identity and multilocus phylogenetic analysis. Genome Announc.

[34] Richter M, Rosselló-Móra R (2009). Shifting the genomic gold standard for the prokaryotic species definition. Proc Natl Acad Sci U S A.

[35] Grant JR, Stothard P (2008). The CGView server: a comparative genomics tool for circular genomes. Nucleic Acids Res.

[36] Seemann T (2014). Prokka: rapid prokaryotic genome annotation. Bioinformatics.

[37] Nawrocki EP, Eddy SR (2013). Infernal 1.1: 100-fold faster RNA homology searches. Bioinformatics.

[38] Hyatt D, Chen G-L, Locascio PF, Land ML, Larimer FW (2010). Prodigal: prokaryotic gene recognition and translation initiation site identification. BMC Bioinformatics.

[39] Laslett D, Canback B (2004). ARAGORN, a program to detect tRNA genes and tmRNA genes in nucleotide sequences. Nucleic Acids Res.

[40] Pruitt KD, Tatusova T, Brown GR, Maglott DR (2012). Ncbi reference sequences (RefSeq): current status, new features and genome annotation policy. Nucleic Acids Res.

[41] Fu L, Niu B, Zhu Z, Wu S, Li W (2012). CD-HIT: accelerated for clustering the next-generation sequencing data. Bioinformatics.

[42] UniProt Consortium (2008). The universal protein resource (UniProt). Nucleic Acids Res.

[43] Page AJ, Cummins CA, Hunt M, Wong VK, Reuter S (2015). Roary: rapid large-scale prokaryote pan genome analysis. Bioinformatics.

[44] Katoh K, Standley DM (2013). MAFFT multiple sequence alignment software version 7: improvements in performance and usability. Mol Biol Evol.

[45] Price MN, Dehal PS, Arkin AP (2009). FastTree: computing large minimum evolution trees with profiles instead of a distance matrix. Mol Biol Evol.

[46] Suchard MA, Lemey P, Baele G, Ayres DL, Drummond AJ (2018). Bayesian phylogenetic and phylodynamic data integration using beast 1.10. Virus Evol.

[47] Darriba D, Taboada GL, Doallo R, Posada D (2012). jModelTest 2: more models, new heuristics and parallel computing. Nat Methods.

[48] Rambaut A, Drummond AJ, Xie D, Baele G, Suchard MA (2018). Posterior Summarization in Bayesian phylogenetics using tracer 1.7. Syst Biol.

[49] Bouckaert R, Heled J, Kühnert D, Vaughan T, Wu C-H (2014). Beast 2: a software platform for Bayesian evolutionary analysis. PLoS Comput Biol.

[50] Letunic I, Bork P (2016). Interactive tree of life (iTOL) V3: an online tool for the display and annotation of phylogenetic and other trees. Nucleic Acids Res.

[51] Huson DH, Bryant D (2006). Application of phylogenetic networks in evolutionary studies. Mol Biol Evol.

[52] Huerta-Cepas J, Szklarczyk D, Forslund K, Cook H, Heller D (2016). eggNOG 4.5: a hierarchical orthology framework with improved functional annotations for eukaryotic, prokaryotic and viral sequences. Nucleic Acids Res.

[53] Jia B, Raphenya AR, Alcock B, Waglechner N, Guo P (2017). Card 2017: expansion and model-centric curation of the comprehensive antibiotic resistance database. *Nucleic Acids* Res.

[54] Zankari E, Hasman H, Cosentino S, Vestergaard M, Rasmussen S (2012). Identification of acquired antimicrobial resistance genes. J Antimicrob Chemother.

[55] Feldgarden M, Brover V, Haft DH, Prasad AB, Slotta DJ (2019). Using the NCBI AMRFinder tool to determine antimicrobial resistance genotype-phenotype correlations within a collection of NARMS isolates. bioRxiv.

[56] Gupta SK, Padmanabhan BR, Diene SM, Lopez-Rojas R, Kempf M (2014). ARG-ANNOT, a new bioinformatic tool to discover antibiotic resistance genes in bacterial genomes. Antimicrob Agents Chemother.

[57] Chen L, Zheng D, Liu B, Yang J, Jin Q (2016). VFDB 2016: hierarchical and refined dataset for big data analysis--10 years on. Nucleic Acids Res.

[58] Carattoli A, Zankari E, García-Fernández A, Voldby Larsen M, Lund O (2014). In silico detection and typing of plasmids using PlasmidFinder and plasmid multilocus sequence typing. Antimicrob Agents Chemother.

[59] Hunt M, Mather AE, Sánchez-Busó L, Page AJ, Parkhill J (2017). ARIBA: rapid antimicrobial resistance genotyping directly from sequencing reads. Microb Genom.

[60] Krasselt M, Baerwald C (2016). Efficacy and safety of modified-release prednisone in patients with rheumatoid arthritis. Drug Des Devel Ther.

[61] Barkia I, Saari N, Manning SR (2019). Microalgae for high-value products towards human health and nutrition. Mar Drugs.

[62] Laudadio I, Fulci V, Palone F, Stronati L, Cucchiara S (2018). Quantitative assessment of shotgun Metagenomics and 16S rDNA amplicon sequencing in the study of human gut microbiome. OMICS.

[63] Lynch SV, Pedersen O (2016). The human intestinal microbiome in health and disease. N Engl J Med.

[64] Gillings MR, Paulsen IT, Tetu SG (2015). Ecology and evolution of the human microbiota: fire, farming and antibiotics. Genes.

[65] Lagier J-C, Dubourg G, Million M, Cadoret F, Bilen M (2018). Culturing the human microbiota and culturomics. Nat Rev Microbiol.

[66] Galperin MY, Brover V, Tolstoy I, Yutin N (2016). Phylogenomic analysis of the family *Peptostreptococcaceae* (Clostridium cluster XI) and proposal for reclassification of *Clostridium litorale* (Fendrich *et al.* 1991) and *Eubacterium acidaminophilum* (Zindel *et al.* 1989) as *Peptoclostridium litorale* gen. nov. comb. nov. and *Peptoclostridium acidaminophilum* comb. nov. Int J Syst Evol Microbiol.

[67] Gerstein AC, Jean-Sébastien M (2011). Small is the new big: assessing the population structure of microorganisms. Mol Ecol.

[68] Kung VL, Ozer EA, Hauser AR (2010). The accessory genome of Pseudomonas aeruginosa. Microbiol Mol Biol Rev.

[69] Motayo BO, Oluwasemowo OO, Olusola BA, Opayele AV, Faneye AO (2019). Phylogeography and evolutionary analysis of African rotavirus a genotype G12 reveals district genetic diversification within lineage III. Heliyon.

[70] Lin L, Zhang J (2017). Role of intestinal microbiota and metabolites on gut homeostasis and human diseases. BMC Immunol.

[71] Fu X, Liu Z, Zhu C, Mou H, Kong Q (2019). Nondigestible carbohydrates, butyrate, and butyrate-producing bacteria. Crit Rev Food Sci Nutr.

[72] Suzuki TA, Worobey M (2014). Geographical variation of human gut microbial composition. Biol Lett.

[73] van Schaik W (2015). The human gut resistome. Philos Trans R Soc Lond B Biol Sci.

[74] Ishikawa J, Chiba K, Kurita H, Satoh H (2006). Contribution of rpoB2 RNA polymerase beta subunit gene to rifampin resistance in Nocardia species. Antimicrob Agents Chemother.

[75] Garcia-Gutierrez E, Mayer MJ, Cotter PD, Narbad A (2019). Gut microbiota as a source of novel antimicrobials. Gut Microbes.

