# Supplementary material for: Comprehensive genome analyses of Sellimonas intestinalis, a potential biomarker of homeostasis gut recovery
Source: Microb Genom. 2020 Nov 18;6(12):mgen000476. doi: 10.1099/mgen.0.000476 (PMC8116674; doi:10.1099/mgen.0.000476)
Supplement: Supplementary material 1 [file mgen-6-476-s001.pdf]

Comprehensive genome analyses of *Sellimonas intestinalis*, a potential biomarker of homeostasis gut recovery

Marina Muñoz[1,2], Enzo Guerrero-Araya[1,2], Catalina Cortés-Tapia[1,2], Ángela Plaza-Garrido[1,2], Trevor D. Lawley[3] and Daniel Paredes-Sabja[1,2,4] *

[1] Microbiota-Host Interactions and Clostridia Research Group, Departamento de Ciencias Biológicas, Facultad de Ciencias de la Vida, Universidad Andrés Bello, Santiago, Chile

[2] ANID – Millennium Science Initiative Program - Millennium Nucleus in the Biology of the Intestinal Microbiota, Santiago, Chile

[3] Host–Microbiota Interactions Laboratory, Wellcome Trust Sanger Institute, Wellcome Genome Campus, Hinxton, United Kingdom

[4] Department of Biology, Texas A&M University, College Station, TX, 77843, USA.

Supplementary material

Supplementary Figure 1. Microscopic morphology of *Sellimonas intestinalis* isolate. The results indicate a Gram-positive bacterial with coccoid morphology.

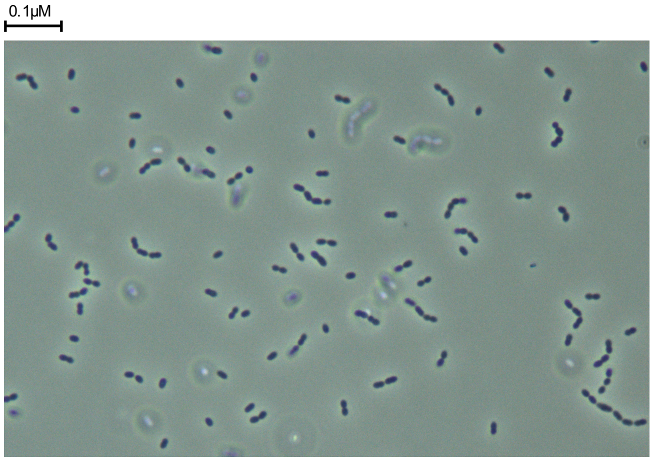

Supplementary Figure 2. Phylogenetic reconstruction of 2,902 publicly available genomes of *Lachnospiraceae* and *Ruminococcacea* members based on 16S-rRNA alignment which allowed to define a node well supported which included the studied assembly and other 9 genomes.

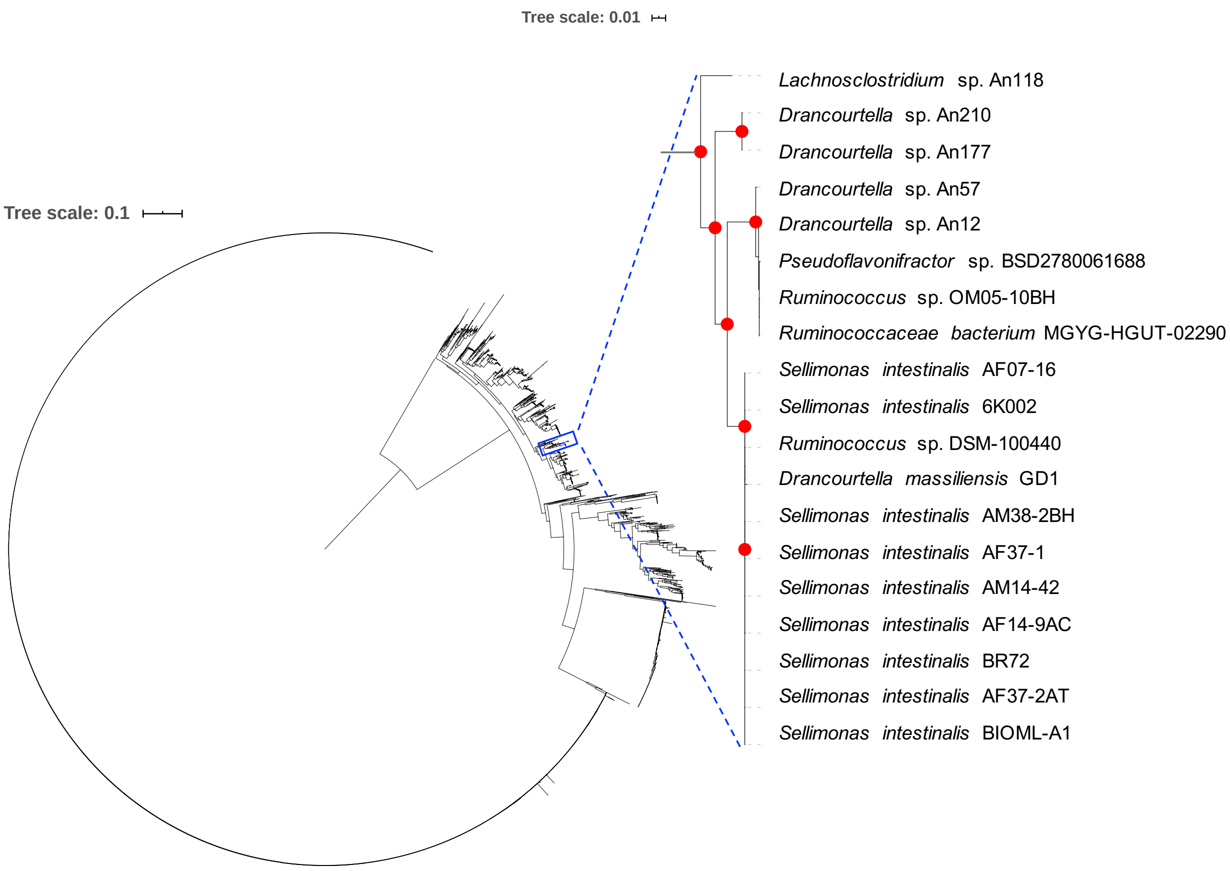

Supplementary Figure 3. Graphical map of the 11 genome assemblies identified as belonging to the same species of studied genome, built in the CGview server [34].

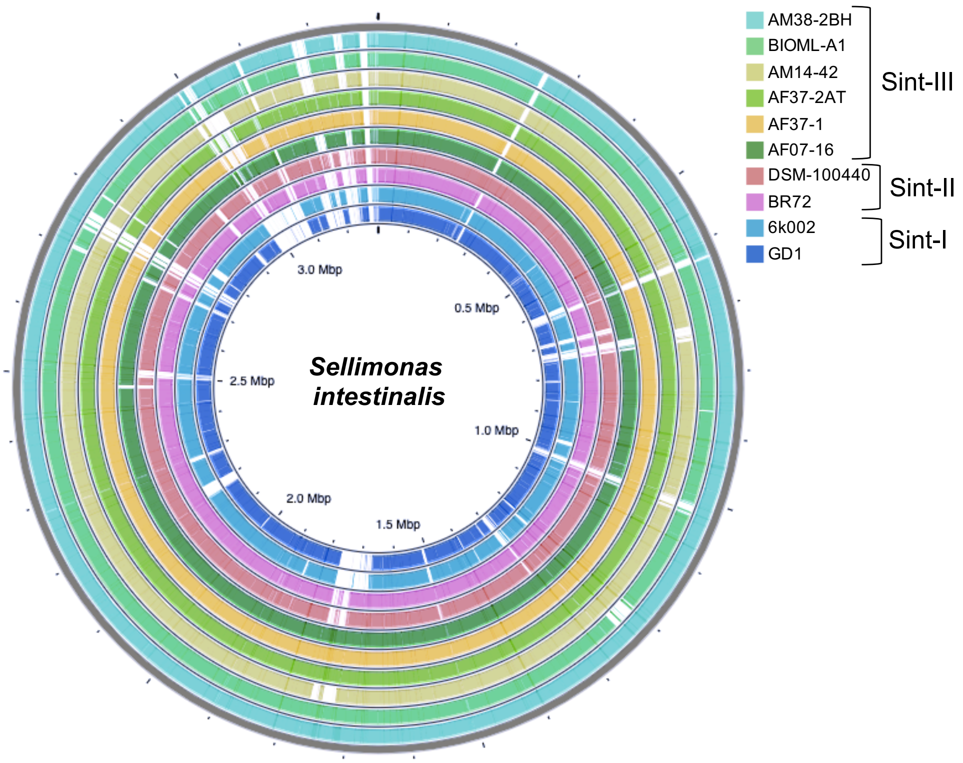

Supplementary Table 1. Comparison of 16S-rRNA sequence using BLAST which revealed that the analyzed genome belongs to one of the following genera: *Ruminococcus*, *Drancourtella* or *Sellimonas*.

Description	Max Score	Total Score	Query Cover	E value	Per. Ident	Accession
Ruminococcus sp. DSM 100440 16S ribosomal RNA gene, partial sequence	2732	2732	97%	0	99.93%	KT156811.1
Uncultured bacterium clone 002-d3 16S ribosomal RNA gene, partial sequence	2697	2697	96%	0	99.86%	DQ904955.1
Drancourtella massiliensis strain GD1 16S ribosomal RNA, partial sequence	2695	2695	97%	0	99.59%	NR_144722.1
Ruminococcus sp. ACB-89 16S ribosomal RNA gene, partial sequence	2571	2571	92%	0	99.64%	KU316944.1
Sellimonas intestinalis strain HF3088 16S ribosomal RNA gene, partial sequence	2555	2555	92%	0	99.64%	MG015712.1

Supplementary Table 2. Information of *S. intestinalis* genomes included in comparative analyses.

**Genome ID**	**1653434.4**	**1653434.5**	**1653434.6**	**1653434.60**	**1653434.7**	**1653434.8**	**1653434.9**
Genome Name	Sellimonas intestinalis strain AM14-42	Sellimonas intestinalis strain AF37-1	Sellimonas intestinalis strain AF37-2AT	Sellimonas intestinalis strain BR72	Sellimonas intestinalis strain AF14-9AC	Sellimonas intestinalis strain AF07-16	Sellimonas intestinalis strain AM38-2BH
Strain	AM14-42	AF37-1	AF37-2AT	BR72	AF14-9AC	AF07-16	AM38-2BH
Completion Date	2018-08-31T00:00:00Z	2018-08-31T00:00:00Z	2018-08-31T00:00:00Z	2015-09-10T00:00:00Z	2018-08-31T00:00:00Z	2018-08-31T00:00:00Z	2018-08-31T00:00:00Z
BioProject Accession	PRJNA482748	PRJNA482748	PRJNA482748	PRJNA283562	PRJNA482748	PRJNA482748	PRJNA482748
BioSample Accession	SAMN09734813	SAMN09734655	SAMN09734663	SAMN03846811	SAMN09734275	SAMN09734193	SAMN09736505
Assembly Accession	GCA_003434665.1	GCA_003435155.1	GCA_003435175.1	GCA_001280875.1	GCA_003435295.1	GCA_003435315.1	GCA_003435335.1
GenBank Accessions	QVLW01000000	QVLY01000000	QVLX01000000	LGAJ01000000	QVMB01000000	QVMC01000000	QVMD01000000
Sequencing Centers	BGI	BGI	BGI	Seoul National University	BGI	BGI	BGI
Sequencing Platform	Illumina Hiseq	Illumina Hiseq	Illumina Hiseq	Illumina MiSeq	Illumina Hiseq	Illumina Hiseq	Illumina Hiseq
Sequencing Depth	100x	100x	100x	207.0x	100x	100x	100x
Assembly Method	SOAPdenovo v. 2	SOAPdenovo v. 2	SOAPdenovo v. 2	CLC Genomics Workbench v. 5.5.1	SOAPdenovo v. 2	SOAPdenovo v. 2	SOAPdenovo v. 2
Contigs	35	34	36	33	36	27	27
Genome Length	3147695	3270028	3262745	3189052	3301306	3206689	3234776
GC Content	45.06748	45.19193	45.19216	45.31911	45.071102	45.167336	45.121597
PATRIC CDS	3235	3401	3393	3237	3435	3294	3323
RefSeq CDS	3009	3163	3154		3198	3077	3100
Isolation Source	feces	feces	feces	stool	feces	feces	feces
Collection Date	2013-08-01	2014-02-01	2014-02-01	13-Mar-2015	2013-10-01	2013-06-01	2014-01-01
Isolation Country	China	China	China		China	China	China
Host Name	Human, Homo sapiens	Human, Homo sapiens	Human, Homo sapiens	Homo sapiens	Human, Homo sapiens	Human, Homo sapiens	Human, Homo sapiens
Genome Quality	Good	Good	Good	Good	Good	Good	Good

Supplementary Table 3. Pangenome analysis of *S. instestinalis* dataset showed a codifying potential of 4,627 genes. The core genes used to population genetic structure and evolutionary analyses are those present in the 11 analyzed genomes (*n*=2,318 genes).

(The file exceeds the number of entries to be transferred to word. Please check the excel file)
